# Supplementary material for: The association of innate and adaptive immunity, subclinical atherosclerosis, and cardiovascular disease in the Rotterdam Study: A prospective cohort study
Source: PLoS Med. 2020 May 7;17(5):e1003115. doi: 10.1371/journal.pmed.1003115 (PMC7205222; doi:10.1371/journal.pmed.1003115)
Supplement: S1 Analysis Plan — (DOCX) [file pmed.1003115.s002.docx]

Statistical analysis plan

INNATE AND ADAPTIVE IMMUNITY, SUBCLINICAL ATHEROSCLEROSIS, AND THE RISK OF CARDIOVASCULAR DISEASE: A PROSPECTIVE COHORT STUDY

Date: January 2019

Version: 2.0

Research question: Are markers of the immune system, i.e. granulocytes, lymphocytes and granulocytes and their derived ratios associated with an increased risk of cardiovascular outcomes?

Exposures: granulocytes, lymphocytes, platelets and derived ratios

Outcomes: Hard atherosclerotic cardiovascular disease (ASCVD): composed of fatal and nonfatal myocardial infarction (MI), other coronary heart disease (CHD) mortality, and stroke. Events assessed until January 1, 2015.

Secondary outcomes:

- hard CHD, composed of fatal and nonfatal MI and CHD mortality.
- Stroke

Baseline: ergo4

Repeated measurements: ergo 5, ergo 6

Timescale: Follow-up

Exclude: previous ASCVD

Stratify: age (65), sex, CRP, lipid-lowering medication

Sensitivity analyses:

- Exclude previous comorbidities: previous cancer other than basal-cell skin cell carcinoma, or ongoing use of systemic anti-inflammatory treatment
- Age as timescale

Not prespecified analyses:

- Stratifying by coronary artery calcification volume (at cut-off of 10 cubic millimetres) and smoking
- A cross-sectional analysis assessing the association of the granulocyte, platelet, and lymphocyte counts, and their derived ratios at baseline per standard deviation increase with calcification volume in each vessel bed (coronary arteries, aortic arch, extracranial carotid arteries, and intracranial carotid arteries) using linear regression models
- A mediation analysis to assess a possible mediating effect of calcification volume in the coronary arteries and intracranial carotid arteries on the association between innate immunity and the risk of CHD and stroke, respectively
