# Supplementary figures and images for: The association of innate and adaptive immunity, subclinical atherosclerosis, and cardiovascular disease in the Rotterdam Study: A prospective cohort study
Source: PLoS Med. 2020 May 7;17(5):e1003115. doi: 10.1371/journal.pmed.1003115 (PMC7205222; doi:10.1371/journal.pmed.1003115)

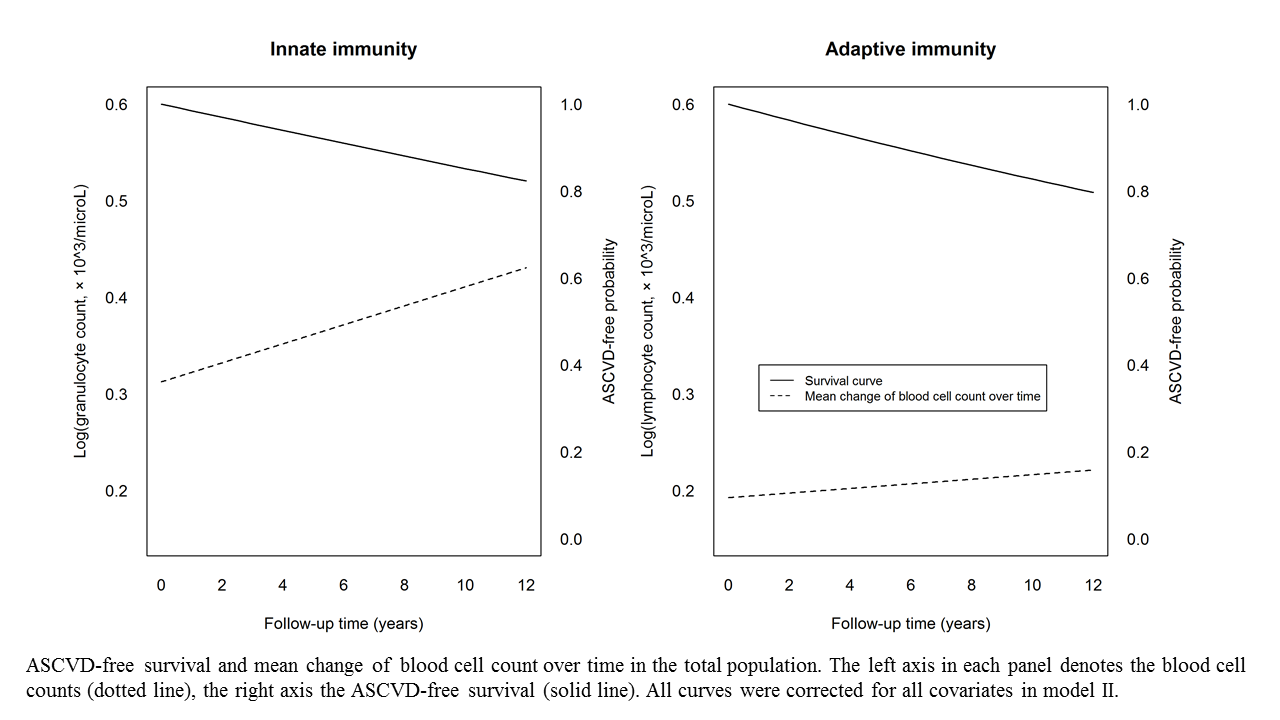

Supplement: S1 Fig — The left axis in each panel denotes the blood cell count (dotted line), the right axis the ASCVD-free survival (solid line). All curves were adjusted for all covariates in model II. (TIF) [file pmed.1003115.s003.tif]

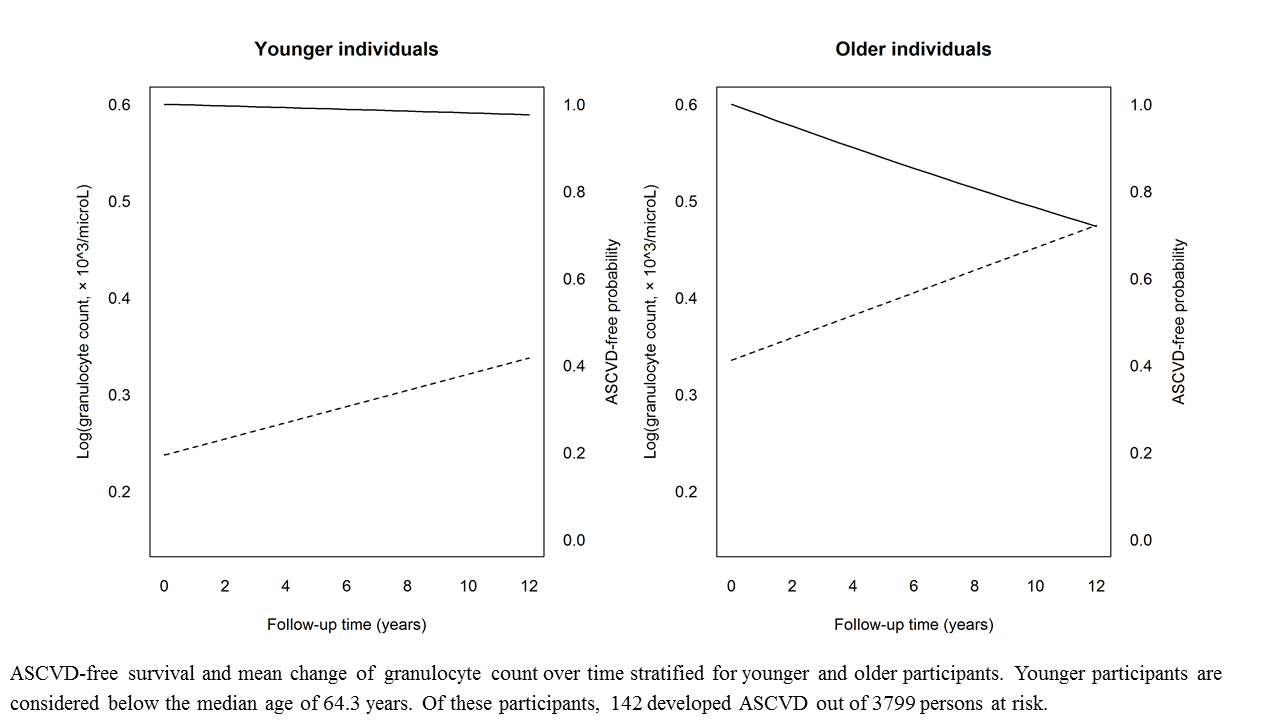

Supplement: S2 Fig — Younger participants are those below the median age of 64 years. Of these participants, 142 developed ASCVD, out of 3,799 persons at risk. (TIF) [file pmed.1003115.s004.tif]
