# Supplementary material for: The association of innate and adaptive immunity, subclinical atherosclerosis, and cardiovascular disease in the Rotterdam Study: A prospective cohort study
Source: PLoS Med. 2020 May 7;17(5):e1003115. doi: 10.1371/journal.pmed.1003115 (PMC7205222; doi:10.1371/journal.pmed.1003115)
Supplement: S1 Table — Adjusted for age, sex, education, smoking status, body mass index, diabetes mellitus, systolic blood pressure, diastolic blood pressure, antihypertensive medication, HDL cholesterol, total cholesterol, and lipid-lowering medication. aAll markers were natural log-transformed (Ln[immunity components × 103/μl]). *Analysis for each blood cell type adjusted for the baseline blood cell counts of the remaining 2 blood cell types. CI, confidence interval; GLR, granulocyte-to-lymphocyte ratio; PLR, platelet-to-lymphocyte ratio; SII, systemic immune-inflammation index. (DOCX) [file pmed.1003115.s005.docx]

**Table S3** Models for the stratified analyses.

| Laboratory assessment^a^ |  | Age |  | Sex |  | Smoking |
| --- | --- | --- | --- | --- | --- | --- |
| Per doubling | *P* for interaction | HR (95% CI), *P* | *P* for interaction | HR (95% CI), *P* | *P* for interaction |  |
| Granulocytes* | 0.002 | Low: 2.32 (1.20-4.47), <.001 | 0.245 | Women: 1.30 (0.88-1.93), 0.18 | 0.565 | Never: 1.49 (1.06-2.09), 0.02 |
|  |  | High: 1.67 (1.21-2.29), 0.002 |  | Men: 2.32 (1.53-3.52), <.001 |  | Ever: 2.19 (1.27-3.77), 0.005 |
| Platelets* | 0.867 | Low: 1.65 (0.87-3.14), 0.13 | 0.167 | Women: 1.11 (0.76-1.62), 0.6 | 0.784 | Never: 1.34 (1.00-1.81), 0.05 |
|  |  | High: 1.08 (0.81-1.45), 0.6 |  | Men: 1.26 (0.88-1.79), 0.21 |  | Ever: 0.79 (0.47-1.31), 0.363 |
| Lymphocytes* | 0.694 | Low: 0.97 (0.56-1.66), 0.9 | 0.209 | Women: 0.83 (0.62-1.11), 0.2 | 0.028 | Never: 0.78 (0.61-0.99), 0.04 |
|  |  | High: 0.87 (0.7-1.09), 0.23 |  | Men: 0.91 (0.68-1.22), 0.55 |  | Ever: 1.06 (0.73-1.55), 0.746 |
| GLR | 0.009 | High: 1.3 (1.08-1.58), 0.007 | 0.945 | Men: 1.49 (1.15-1.93), 0.003 | 0.172 | Never: 1.38 (1.12-1.7), 0.003 |
|  |  | Low: 1.52 (0.93-2.48), 0.09 |  | Women: 1.25 (0.96-1.62), 0.1 |  | Ever: 1.28 (0.85-1.93), 0.241 |
| PLR | 0.8 | High: 1.16 (0.97-1.38), 0.11 | 0.966 | Men: 1.19 (0.95-1.49), 0.13 | 0.009 | Never: 1.34 (1.1-1.62), 0.003 |
|  |  | Low: 1.29 (0.77-1.87), 0.41 |  | Women: 1.17 (0.92-1.49), 0.21 |  | Ever: 0.82 (0.61-1.1), 0.181 |
| SII | 0.641 | High: 1.14 (0.98-1.32), 0.09 | 0.601 | Men: 1.27 (1.05-1.53), 0.02 | 0.238 | Never: 1.22 (1.04-1.43), 0.016 |
|  |  | Low: 1.40 (0.96-2.04), 0.08 |  | Women: 1.11 (0.90-1.37), 0.32 |  | Ever: 1.06 (0.79-1.43), 0.705 |
|  |  | Calcifications |  | CRP |  | Lipid-lowering medication |
| Granulocytes* | 0.559 | Low: 3.51 (1.14-10.83), 0.03 | 0.488 | Low: 1.47 (0.8-2.71), 0.21 | 0.798 | Yes: 1.77 (0.89-3.49), 0.10 |
|  |  | High: 1.86 (1.05-3.31), 0.03 |  | High: 0.94 (0.5-1.76), 0.84 |  | No: 1.97 (1.45-2.68), <.001 |
| Platelets* | 0.178 | Low: 0.56 (0.19-1.65), 0.29 | 0.806 | Low: 1.35 (0.76-2.41), 0.31 | 0.057 | Yes: 0.69 (0.39-1.24), 0.21 |
|  |  | High 1.11 (0.68-1.81), 0.69 |  | High: 1.07 (0.62-1.83), 0.82 |  | No: 1.26 (0.95-1.67), 0.12 |
| Lymphocytes* | 0.073 | Low: 2.22 (1.04-4.71), 0.04 | 0.176 | Low: 0.79 (0.49-1.27), 0.33 | 0.605 | Yes: 0.86 (0.53-1.40), 0.54 |
|  |  | High: 0.73 (0.50-1.09), 0.13 |  | High: 0.79 (0.54-1.16), 0.22 |  | No: 0.93 (0.74-1.16), 0.51 |
| GLR | 0.354 | Low: 1.03 (0.51-2.08), 0.93 | 0.925 | Low: 1.33 (0.92-1.94), 0.13 | 0.707 | Yes: 1.34 (0.88-2.06), 0.18 |
|  |  | High: 1.55 (1.08-2.24), 0.02 |  | High: 1.15 (0.83-1.59), 0.40 |  | No: 1.41 (1.15-1.74), 0.001 |
| PLR | 0.018 | Low: 0.54 (0.29-1.02), 0.06 | 0.45 | Low: 1.22 (0.9-1.65), 0.21 | 0.315 | Yes: 0.97 (0.67-1.41), 0.88 |
|  |  | High: 1.29 (0.94-1.77), 0.12 |  | High: 1.22 (0.9-1.65), 0.21 |  | No: 1.17 (0.97-1.41), 0.09 |
| SII | 0.176 | Low: 0.91 (0.51-1.62), 0.75 | 0.81 | Low: 1.24 (0.92-1.68), 0.16 | 0.396 | Yes: 0.99 (0.72-1.36), 0.94 |
|  |  | High: 1.25 (0.95-1.64), 0.11 |  | High: 1.06 (0.81-1.37), 0.69 |  | No: 1.23 (1.05-1.44), 0.009 |

Abbreviations: CI = confidence interval; GLR = granulocyte-to-lymphocyte ratio; PLR = platelet-to-lymphocyte ratio; SII = systemic immune-inflammation index. Adjusted for age, sex, education, smoking status, body mass index, diabetes mellitus, systolic blood pressure, diastolic blood pressure, blood pressure lowering medication, HDL cholesterol, total cholesterol and lipid lowering medication. *Inflammatory markers corrected for two remaining blood cell types. ^a^All markers were natural log-transformed (Ln[immunity components × 10^3^/microL]).
