# Supplementary material for: The association of innate and adaptive immunity, subclinical atherosclerosis, and cardiovascular disease in the Rotterdam Study: A prospective cohort study
Source: PLoS Med. 2020 May 7;17(5):e1003115. doi: 10.1371/journal.pmed.1003115 (PMC7205222; doi:10.1371/journal.pmed.1003115)
Supplement: Attachment — Submitted filename: fani.pdf [file pmed.1003115.s006.pdf]

This paper reports association between immunity markers and cardiovascular disease in the Rotterdam Study.

I was asked for a statistical report and I interpret that to include all aspects of the design and conduct of the study.

Points of detail

Pages 6 and 7 Strictly speaking the numbers of people reported here are part of the results. The authors did not plan, for instance, to have 8 people have incomplete laboratory assessment.

Page 10 If I calculate it correctly $\frac{0.05}{12 \cdot 6} = 0.00069$ not 0.001. As far as I can see these interactions are not reported in detail anywhere. The supplement would seem appropriate and both adjusted and unadjusted would be useful.

Page 12 The authors have already told us on page 11 that they considered 0.05 as their critical level for statistical significance so why does 0.71 to 1.07 count? This is also repeated elsewhere including the abstract.

Page 13 I am not sure it is necessary to repeat all the numbers which are in Table 2 but if the authors want to do that then they should also copy the results for lymphocytes throughout.

Page 13 It would be much better to express the confidence intervals throughout as either [-10% to 32.2%] or [-10%, 32.2%] to avoid ambiguity. I know that the authors have used a minus sign and a dash in their version but they are too similar visually

Page 15 Now we finally have it confirmed that the lymphocyte results were not statistically significant.

Figures S1 and S2 Are these really survival curves? They look very smooth to be Kaplan–Meier even given the numbers involved.

Points of more substance

The interactions

Performing Bonferroni correction for the whole set of interactions simultaneously seems to me very conservative. If I read page 7 correctly some of the

six markers are derived from others so can hardly be called independent. I suspect the effect modifiers are related as well. Given that interactions are usually less precisely estimated anyway the authors' approach here seems to me almost designed to ensure that the interactions can be dismissed. If there is any existing scientific hypothesis about any of these interactions then it would be better to report that in full unadjusted. If there is no hypothesis then perhaps adjusting only for smaller sets of interactions would be better. Without any clinical knowledge in this area I cannot suggest whether it would be better to do this by effect modifier or by marker.

Statistical significance

The statements the authors make about lymphocytes are true: they are associated with a decrease in risk. Stating that though without any qualification about the precision of that estimate is misleading. I am no great advocate of arbitrary significance levels but one should be consistent.

Summary

Points for clarification and some suggestions about the interactions.

Michael Dewey
