# Supplementary material for: The association of innate and adaptive immunity, subclinical atherosclerosis, and cardiovascular disease in the Rotterdam Study: A prospective cohort study
Source: PLoS Med. 2020 May 7;17(5):e1003115. doi: 10.1371/journal.pmed.1003115 (PMC7205222; doi:10.1371/journal.pmed.1003115)
Supplement: Attachment — Submitted filename: Authors reply_20200226.docx [file pmed.1003115.s007.docx]

## Authors’ response to reviewers

**Manuscript title**: *Innate and adaptive immunity, subclinical atherosclerosis, and the risk of cardiovascular disease: a prospective cohort study*. Manuscript # PMEDICINE-D-19-03937

We thank the editors and reviewers for their thoughtful comments and feedback. We have taken careful notice of the comments and have critically revised our manuscript. All revisions in the manuscript are indicated by their specific line numbers for your convenience (referring to the document with tracked changes).

**Editors' comments:**

1. *Please include line numbers in your revision.*

**Response**: We have included line numbers in the revision as requested.

2. *We suspect that your study reports a retrospective analysis of a prospectively gathered dataset. Therefore, please remove the word "prospective" from your title; we suggest as an alternative "Innate and adaptive immunity, subclinical atherosclerosis and risk of cardiovascular disease: a cohort study."*

**Response**: As suggested by the editors we have changed the title to: *"Innate and adaptive immunity, subclinical atherosclerosis and risk of cardiovascular disease: a cohort study."* (page 1, lines 1-2).

3. *At the start of your abstract, please add an introductory sentence, say, to provide some background as to why the study was conceived.*

**Response:** We have now extended the abstract as follows: *Background: Atherosclerotic cardiovascular disease (ASCVD) is driven by multifaceted contributions of the immune system. However, the dysregulation of immune cells that leads to ASCVD is poorly understood. We determined the association of components of innate and adaptive immunity longitudinally with ASCVD, and assessed whether arterial calcifications play a role in this association.* (page 2, lines 28-33).

4. *Please combine the "methods" and "findings" subsection of your abstract. The final sentence of the new combined subsection should summarize the study's main limitations.*

**Response**: We have combined the “methods” and “findings” subsection of our abstract as requested by the editors. We also added a final sentence to this section stating the limitations: *“A limitation of our study is that studying the etiology of ASCVD remains difficult within an epidemiological setting due to the limited availability of surrogates for innate and especially adaptive immunity.”* (page 2-3, lines 50-52).

5. *Early in the "methods and findings" subsection of your abstract, please use the passive voice (e.g., "... granulocyte and lymphocyte counts were determined ..." [stating interval of data collection]).*

**Response**: We changed the beginning of the “methods and findings” subsection as suggested to: “*Granulocyte (innate immunity) and lymphocyte counts (adaptive immunity) were repeatedly determined [2002-2015] in ASCVD-free participants of the population-based Rotterdam Study.”* (page 2, lines 34-36).

6. *To commence the "conclusions" subsection of your abstract, please begin the sentence "In this study, we found that an increased granulocyte count was associated with a higher risk of ...", or similar.*

**Response:** We modified the beginning of the conclusion section to the following: “*In this study, we found that an increased granulocyte count was associated with a higher risk of ASCVD whereas an increase in lymphocytes was protective for ASCVD in the general population. Moreover, higher levels of granulocytes were associated with larger volume of arterial calcification. Arterial calcifications may explain a proportion of the link between granulocytes and ASCVD.”* (page 3, lines 53-57).

7. *Noting the observational design of your study, please avoid language which implies causality (e.g., in the abstract, "The effect of granulocytes on incident CHD ...").*

**Response:** As suggested by the editors, we have modified language which implies causality, namely in the abstract: *“The association between granulocyte counts and incident CHD and stroke was partly mediated by”* (page 2, lines 47-48), in the methods section: *“We chose the innate immunity marker displaying the strongest association with both calcifications and incident CHD or stroke for this analysis.”* (page 14, lines 276-277), in the results section: *“The association between granulocytes and incident CHD was partly mediated by coronary artery calcifications (overall proportion mediated [95%CI]: 19.0% [-10% to 32.3%]), while the association between granulocytes and incident stroke..”* page 18, lines 351-353) and discussion section: *“Coronary artery calcifications mediated 19.0% of the association between granulocytes and CHD, while intracranial artery calcifications mediated 14.9% of the association between granulocytes and stroke.”* page 19, lines 362-364.

8. *In the abstract and elsewhere, please add p values alongside 95% CI where available.*

**Response:** We have stated *P­-*values alongside 95% confidence intervals as requested by the editors.

9. *After your abstract, please add a new and accessible "author summary" section in non-identical prose. You may find it helpful to consult one or two recent research articles published in PLOS Medicine to get a sense of the preferred style.*

**Response:** We have added an author summary to page 4 of our manuscript as requested by the editors.

10. *Early in the methods section, please state whether your study had a protocol or prespecified analysis plan, and if so attach the relevant document(s) as a supplementary file (referred to in the text). Please highlight analyses that were not prespecified.*

**Response:** We thank the editors for the suggestion and agree that adding our analysis plan increases transparency on the conception and execution of the study. We have therefore added our prespecified analysis plan to the supplementary materials and we now refer to the plan early in the methods section: *“For the current study, the analysis plan was drafted in January 2019 (S1 Analysis Plan).”* (page 8, lines 126-127). Within this plan, we have mentioned the analyses that were not prespecified.

11. *In the paragraph discussing study limitations in the discussion section of your main text, we suggest noting the possible influence of unmeasured confounders.*

As suggested by the editor, we have now mentioned unmeasured confounders in the limitations section of the discussion: *“Fourth, it is important to note the possible influence of unmeasured confounders on our results.”* (page 23, lines 469-470). We would like to mention that we reconsidered our joint model adjustments and also added a continuous adjustment for diastolic blood pressure to decrease confounding bias. That is why some of the joint modelling estimates could have changed throughout the manuscript.

12. *Throughout the text, please format reference call-outs as follows: "... neurodegenerative disorders [9,10], but also ...".*

As suggested by the editors we have formatted reference call-outs throughout the text, to match the journal’s reference style (point 14 below).

13. *Please remove trade marks from the paper.*

We have removed all trademarks from the paper as requested by the editors, more specifically on page 9, lines 147-148: *“Full blood count measurements were performed using the COULTER Ac·T diff2 Hematology Analyzer”.*

14. *Please revisit your reference list to ensure that all reference citations match journal style. Italics should be converted to plain text; where appropriate, 6 author names should be listed, followed by "et al."; please remove "and" from lists of author names; and please ensure that all references include full access details (e.g., references 8, 14, and 34).*

We have changed the reference style to match the journal.

15. *Please attach a completed checklist for the most appropriate reporting guideline, which we suspect will be STROBE, as a supplementary document (referred to in the methods section). In the checklist, individual items should be referred to by section (e.g., "Methods") and paragraph number rather than by page or line numbers, as the latter generally change in the event of publication.*

We have attached the completed STROBE checklist as a supplementary document and we have referred to it in the Methods section: “*This study is reported as per the Strengthening the reporting of observational studies in epidemiology (STROBE) guideline (S2 STROBE Checklist).*” (page 8, lines 129-130).

**Reviewer #1**

*This paper reports association between immunity markers and cardiovascular disease in the Rotterdam Study. I was asked for a statistical report and I interpret that to include all aspects of the design and conduct of the study.*

***Points of detail***

1. *Pages 6 and 7: Strictly speaking the numbers of people reported here are part of the results. The authors did not plan, for instance, to have 8 people incomplete laboratory assessment.*

**Response:** We have moved the section describing the exclusion of participants to the beginning of the results section: “*From 8,712 participants, we excluded those with a history of ASCVD (n=819) at baseline, participants who had incomplete data regarding their history of ASCVD (n=95), and those who did not provide informed consent to assess medical records during follow-up (n=60). Lastly, we excluded participants with incomplete laboratory assessment (n=8), resulting in 7,730 participants for analysis (Flowchart in* ***Figure 1****). From the fourth round of RS-I and the second round of RS-II, a random sample was invited to undergo non-contrast computed tomography (CT) to quantify arterial calcification. Of these participants, 2,366 had complete calcification and blood assessment.”* (page 16, lines 294-301).

2. *Page 10: If I calculate it correctly 0.05/12*6=0.00069 not 0.001. As far as I can see these interactions are not reported in detail anywhere. The supplement would seem appropriate and both adjusted and unadjusted would be useful.*

**Response:** As also suggested in point 8, we have chosen to omit the Bonferroni correction from our study, due to the conservativeness. We furthermore agree with the reviewer to show the interactions in the supplement. We refer to the interactions on page 17, line 329: “*Results of all stratified analyses with interaction terms are shown in Table S3.*”

3. *Page 12: The authors have already told us on page 11 that they considered 0.05 as their critical level for statistical significance so why does 0.71 to 1.07 count? This is also repeated elsewhere including the abstract.*

**Response:** We have adjusted the statements about lymphocytes throughout the manuscript by adding a qualification about the precision of the estimate as follows: “*while lymphocytes were associated with a decreased ASCVD risk, although not statistically significant (HR[95%CI]: 0.87 [0.71–1.07])*.” in the abstract (page 2, lines 43-45), “*With respect to adaptive immunity, increasing levels of lymphocytes were associated with a decreased risk of ASCVD, although not statistically significant (adjusted HR for doubling lymphocyte count [95% CI] = 0.87 [0.71–1.07])*.” (page 14, lines 276-279) in the results and “*An increase in lymphocytes showed similar protective effects for both CHD and stroke, although not statistically significant (0.88 [0.66–1.17], P=0.37 for CHD and 0.85 [0.63-1.15] for stroke)*.” in the results section (page 15, lines 297-298).

4. *Page 13: I am not sure it is necessary to repeat all the numbers which are in Table 2, but if the authors want to do that then they should also copy the results for lymphocytes throughout.*

**Response:** We have added the numbers from table 2 describing the association between lymphocytes and risk of CHD and stroke: “*An increase in lymphocytes showed similar protective effects for both CHD and stroke, although not statistically significant (0.88 [0.66–1.17], P=0.37 for CHD and 0.85 [0.63-1.15] for stroke)*.” in the results section (page 15, lines 297-298).

5. *Page 13: It would be much better to express the confidence intervals throughout as either [-10% to 32.2%] or [-10%, 32.2%] to avoid ambiguity. I know that the authors have a used a minus sign and a dash in their version but they are too similar visually.*

**Response:** We thank the reviewer for the suggestion and have changed the expression of the confidence intervals to avoid ambiguity for the cross-sectional and mediation analyses (page 16, lines 313, 316, 317, 324 and 325.

6. *Page 15: Now we finally have it confirmed that the lymphocyte results were not statistically significant.*

**Response:** We agree with the reviewer that we should be consistent in stating clearly that the results for lymphocytes did not reach statistical significance in our study, as we have done in response to point 3.

7. *Figure S1 and S2: Are these really survival curves? They look very smooth to be Kaplan-Meier even given the numbers involved.*

**Response:** These are indeed survival curves and not Kaplan-Meier curves. The presented survival curves are corrected for all included covariates (model II). We have clarified this on page 29 by adding the following: “All curves were corrected for all covariates in model II.”

***Points of more substance***

8. ***The interactions****: Performing Bonferroni correction for the whole set of interactions simultaneously seems to me very conservative. If I read page 7 correctly some of the six markers are derived from others so can hardly be called independent. I suspect the effect modifiers are related as well. Given that interactions are usually less precisely estimated anyway the author’s approach here seems to me almost designed to ensure that the interactions can be dismissed. If there is any existing scientific hypothesis about any of these interactions then it would be better to report that in full unadjusted. If there is no hypothesis then perhaps adjusting only for smaller sets of interactions would be better. Without any clinical knowledge in this area I cannot suggest whether to do this by effect modifier or marker.*

**Response:** We agree with the reviewer that Bonferroni correction may be too conservative, especially considering that both markers and effect modifiers are indeed not independent from each other. Therefore, we chose to leave out the Bonferroni correction from the manuscript and show all interactions in the supplement as suggested in point 2.

***Summary:*** *Points for clarification and some suggestions about the interactions.*

**Response:** We thank prof. dr. Michael Dewey for the helpful feedback.

**Reviewer #2**

*I read with great interest the manuscript by Fani et al. regarding associations of innate and adaptive immunity with incident myocardial infarction and stroke. The methods appear to be sound and the manuscript is generally well-written. I have a few major concerns/questions as well as a few minor questions.*

1. *The mediation analysis presents large effect sizes, but they are not statistically significant. Based on the confidence interval, they do not appear to be marginally significant. What is your justification for making a strong claim in the conclusion about a mediating effect when you lack such statistical precision? The assessed association between immunity and calcification is cross-sectional and subject to reverse causality. How do we know it’s not the calcification that leads to higher granulocytes? At a minimum, the potential for reverse causality should be mentioned in the limitations section of the discussion. In the conclusion of the abstract, “Arterial calcifications explain a substantial proportion” may be overstated given the lack of statistical significance and cross-sectional nature of the immunity and calcification association.*

**Response:** We agree with the reviewer on all points. Since the results of our mediation analyses are indeed not statistically significant, we have changed the conclusion into: *“Arterial calcifications may explain a proportion of the link between granulocytes and ASCVD.”* (page 3, lines 58-59). In addition, we have addressed the possibility of reverse causality in the discussion section: “*Fifth, due to the cross-sectional nature of the analyses regarding the association between immunity markers and calcifications, there is potential for reverse causality*.” (page 23, lines 471-473).

2. *I would like to see more discussion on the variability and determinants in the measurement of granulocytes, lymphocytes and platelets. How much does any individuals cell count vary from day to day or year to year? I know acute and chronic infections can lead to significant changes in blood cell counts. Could you potentially be measuring an underlying infectious process that may trigger an ASCVD event? Did you consider a sensitivity analysis excluding ASCVD events within the first year of follow-up?*

**Response:** The reviewer rightfully states that blood cell count measurements are variable, but we do not expect that this variability will affect our results for several reasons. First, we expect to capture a more chronic/stable effect of immune status because we are using blood cell counts measured at multiple time points over years per participant. Although this longitudinal information is collected intermittently and with error, we can successfully reconstruct the complete longitudinal history by postulating a suitable mixed-effects model to describe subject-specific time evolutions. The mixed model then accounts for the measurement error problem by postulating that the observed level of the blood cell count measurements equals the true level of the blood cell count measurements plus a random error term. So the joint model we have used accounts for measurement error or variability of the blood cell count measurements. Second, as a sensitivity analysis we additionally excluded participants with a history of cancer or a history of immune-modulating medication at baseline and we performed additional adjustments for NSAID use. We performed these sensitivity analyses specifically to deal with participants that would have significantly altered blood cell counts due to underlying disease processes. The results of these analyses do not show significant changes in our effect estimates. Lastly, by extensively correcting for potential confounders such as BMI, we try to rule out effects of determinants of blood cell counts as much as possible, but as the editors have rightfully stated, we cannot rule out any residual, unmeasured confounding.

We have added above points to the discussion section just before the limitations section as a separate paragraph: *“Blood cell count measurements display short-term variability or measurement error, but we do not expect that this variability affected our results for several reasons. First, we expect to capture a more chronic or stable effect of immune status because we are using blood cell counts measured at multiple time points per participant. Although this longitudinal information was collected intermittently and with error, we could successfully reconstruct the complete longitudinal history by postulating a suitable mixed-effects model to describe subject-specific time evolutions. The mixed model then accounts for the measurement error problem by postulating that the observed level of the blood cell count measurements equals the true level of the blood cell count measurements plus a random error term. Thus the joint models we have used accounted for measurement error or variability of the blood cell count measurements. Second, as a sensitivity analysis we additionally excluded participants with a history of cancer or a history of immune-modulating medication at baseline and we performed additional adjustments for NSAID use. We performed these sensitivity analyses specifically to deal with participants that would have significantly altered blood cell counts due to underlying disease processes. Results of these analyses did not show significant changes in our effect estimates. Lastly, by extensively correcting for potential confounders such as BMI, we attempted to rule out effects of determinants of blood cell counts as much as possible.”* (page 22, lines 447-463).

3. *In the analysis presented in table 3, did you consider a sensitivity analysis looking at markers and continuous arterial calcification in those only with non-zero arterial calcification? The magnitude of 0 to 1 for volume may not be the same as other incremental increases in arterial calcification.*

**Response:** We have added the requested sensitivity analysis to the manuscript, stating the analysis in the statistical analysis section: *“As a sensitivity analysis, we looked at the association between the granulocyte, platelet, and lymphocyte counts, and their derived ratios and continuous arterial calcification in those only with non-zero arterial calcification, since the magnitude of 0 to 1 for volume may not be the same as other incremental increases in arterial calcification.”* (page 14, lines 272-276) and results section: “Our sensitivity analyses with non-zero calcifications similarly showed associations between higher levels of granulocytes and larger calcification volumes in all arteries, but the analysis with extracranial internal carotid artery calcification as outcome did not reach statistical significance.” (page 18, lines 358-362). We have now summarized the results of these analyses in one sentence, but if the reviewer prefers showing all the results in the supplements, then we would be happy to do so.

4. *For the interaction analyses, I would like to see the effect size (confidence interval) for those older than age 64. Given the very low p-value chosen, I would be curious to see the effect estimates for all the interaction analyses tested. There could be meaningful information in these analyses that would warrant at least a place in the supplement section. Given such a profound effect modification by age, I would like the authors to address/hypothesize at least briefly in the discussion how they interpret such findings.*

**Response:** As also suggested by Reviewer #1 (point 2), we have decided to show all effect sizes and confidence intervals for the stratified analyses in the supplements. We have added our interpretation of the effect modification by age that we observe in the discussion section: *“An interesting finding of our study is that the association between higher levels of granulocytes and risk of ASCVD was more pronounced in younger individuals compared to those aged above the median age of 64 years (with younger age: adjusted HR, [95% CI] for doubling granulocyte count = 2.32 [1.20-4.47], P <.001, with older age: 1.67 [1.21-2.29], P= 0.002). Recently, two studies found that while there is a decline in mortality from acute stroke, stroke event rates and mortality risk in young adults have increased and remained elevated, respectively [44, 45]. This suggests that stroke awareness and stroke prevention need to start at much lower age to reduce the occurrence of stroke in younger people. Our results show that inflammation reduction could be a potential important strategy to achieve this.”* (page 21, lines 420-429).

5. *Methods: Arterial calcification- I would like to see a little more description of how calcified volume was measured instead of having to refer to multiple previous publications. For instances, did you use a summation of pixels? Were there differences in CT scanners used?*

**Response:** As suggested by the reviewer we have extended the description of the calcifications measurements to the following: “*Non-contrast CT images were obtained using a 16-slice or 64-slice multidetector CT scanner (Somatom Sensation 16 or 64; Siemens, Forchheim, Germany). Using a cardiac scan and an extracardiac scan that reached from the aortic root to the intracranial vasculature (1 centimeter above the sella turcica), we visualized the following vessels: coronary arteries, aortic arch, extracranial internal carotid arteries, and intracranial internal carotid arteries. Radiation dose and other imaging parameters of both scans are described elsewhere [24, 25].*

*The amount of calcification in the coronary arteries, aortic arch, and extracranial internal carotid arteries was quantified using widely used, dedicated commercially available software (Syngo.via, Siemens, Germany). Calcification in the coronary arteries comprised a summation of calcification in the left main, left anterior descending, left circumflex and the right coronary artery. Calcification in the aortic arch was scored from the origin of the aortic arch (defined as the image in which the ascending and descending aorta merge into the inner curvature of the aortic arch) to the first 1 cm of the branches originating from the arch. Calcification in the extracranial carotid arteries was assessed within 3 cm proximal and distal of the bifurcation on both sides and summed.*

*Intracranial internal carotid artery calcification was assessed from the horizontal segment of the petrous internal carotid artery to the top of the internal carotid artery. Scoring was done semi-automatically, because automated software is not yet available for this region (the close relationship between calcification and the skull base precludes the use of commercial software given a high false-positive rate). Regions of interest were manually drawn in the course of the intracranial internal carotid artery on both sides after which the number of pixels within these regions above 130 Hounsfield Units was summed and multiplied by the pixel-size, and the slice increment in order to obtain a volume in mm3 [26, 27].”* (pages 10-11, lines 168-192)

24.      Elias-Smale SE, Wieberdink RG, Odink AE, Hofman A, Hunink MG, Koudstaal PJ, et al. Burden of atherosclerosis improves the prediction of coronary heart disease but not cerebrovascular events: the Rotterdam Study. Eur Heart J. 2011;32(16):2050-8. PubMed PMID: 21606087.

25.      Odink AE, van der Lugt A, Hofman A, Hunink MG, Breteler MM, Krestin GP, et al. Risk factors for coronary, aortic arch and carotid calcification; The Rotterdam Study. J Hum Hypertens. 2010;24(2):86-92. PubMed PMID: 19494836.

26.      de Weert TT, de Monye C, Meijering E, Booij R, Niessen WJ, Dippel DW, et al. Assessment of atherosclerotic carotid plaque volume with multidetector computed tomography angiography. Int J Cardiovasc Imaging. 2008;24(7):751-9. PubMed PMID: 18373211.

27.      Bos D, van der Rijk MJ, Geeraedts TE, Hofman A, Krestin GP, Witteman JC, et al. Intracranial carotid artery atherosclerosis: prevalence and risk factors in the general population. Stroke. 2012;43(7):1878-84. PubMed PMID: 22569939.

6. *What is the definition of an immune-modulating medication?*

**Response:** We defined immune-modulating medication as use of NSAIDs [ATC-ode M01]; immunosuppressives [ATC-code L04] plus methotrexate [ATC-code L01BA01]. We have added this description to the methods section, page 11, lines 208-210.

7. *Did you consider a test for non-linearity? The results may be driven by the extremes of the distribution. Consider showing a figure of the dose-response association of blood cell counts with incident ASCVD.*

**Response:** This is indeed a relevant point: Since the survival function *S* depends on the whole history of the true marker levels (*M*), it is important to obtain a good estimate of *M* for an accurate estimation of *S*. This entails considering an elaborate specification of the time structure in the known design matrices in the linear mixed model. After careful examination of possible non-linearity, we found that log transforming our exposure variables provided the best solution to deal with non-linearity, due to the skewed nature of the exposure variables which untransformed could indeed cause problems by the extremes of the distribution. Further examination showed no highly non-linear longitudinal trajectories. Since we are adjusting for covariables and to enhance interpretability of our models, we continued with the log-transformed exposure variables, and keeping the models parsimonious without for example entering natural cubic splines. This reduces the risk of overfitting our models and convergence problems. We have added a phrase to the statistical analysis section to clarify this reasoning to the reader: “*Because the blood-cell counts and their derived ratios have a skewed distribution and to adhere to the linearity assumptions, analysis was based on the natural logarithmic (Ln) transformation of their values*.” (page 12, lines 215-216).

8. *Did you test for the proportional hazard assumption for Cox modelling?*

**Response:** We have indeed tested the proportional hazards assumption using Schoenfeld residuals. We have added the inspection of the Schoenfeld residuals to the manuscript: “*We assessed the joint model assumptions by examining the proportional hazards assumption using Schoenfeld residuals and goodness-of-fit using Cox-Snell residuals for the survival part and marginal residuals for the longitudinal part*.” (page 13, lines 246-248). In the results section, we have added the following: “We observed no major violations of the joint model assumptions.” (page 17, lines 322).

9. *What is the rationale for using the Bonferroni correction in the interaction analysis, but not in your main analysis? If I am not mistaken, typically the p-value for exploratory tests of multiplicative interaction are less stringent than the main analyses. Using such a low p-value for interaction testing may miss important effect modification.*

**Response:** We agree with the reviewer that Bonferroni correction may be too conservative, especially considering that both markers and effect modifiers are indeed not independent from each other. Therefore, in line with point 8 of reviewer #1, we chose to disregard the Bonferroni correction from our analyses and we now show all interaction terms in Table S3.

*Minor comments/suggestions:*

10. *Abstract: In results section the last sentence I believe should have a “, respectively” at the end.*

We have added a “respectively” to the sentence, but at the beginning of the sentence so that the reader does not have to read the sentence again: “*Respectively, the association between granulocyte counts and incident CHD and stroke was partly mediated by coronary artery calcification (19.0%) and intracranial artery calcifications (14.9%)*.” (page 2, lines 47-49).

11. *Abstract: Please report the confidence intervals for the mediation analysis effect sizes.*

**Response:** We have added the confidence intervals as suggested by the reviewer to the mediation analysis estimates.

12. *Statistical analysis- “Results can be interpreted as: A doubling of blood cell counts over time in a person compared to another person gives a HR for the risk of ASCVD.” Is this a doubling of log-transformed cell counts? If so, this is a different scale and should be specified in the text and tables. For the linear regression analysis, you report per standard deviation increase while in the cox models you report a per doubling. Was there a reason to use standardized coefficients for one and a doubling for the other?*

**Response:** The abovementioned interpretation is not a doubling in log-transformed cell counts, but truly a doubling in (unlogged) blood cell counts as mentioned in the methods section. This is because we are using log-log models: log*Y_i_ = α* + *β*log*X*_i_ + *ε*_i_, because both the dependent variable (ASCVD) and independent variables (blood cell counts) are log-transformed variables. The interpretation is given as an expected percentage change in *Y* when *X* increases by some percentage. So in terms of effects of changes in *X* on *Y* (both unlogged): To get the proportional change in *Y* associated with a *p* percent increase in *X,* we can calculate *α* = log([100 + *p*]/100) and take *exp(α * B̂)*. So for a doubling of X (100% increase in *X), α* = log([100 + 100]/100) = log(200/100) = log(2). Log refers to natural logarithms (in the manuscript mentioned as Ln). Following *exp(α * B̂)*, we obtain our hazard ratios by exponentiating Ln(2) times the estimated coefficients from the Cox model as described in our manuscript. To clarify this, we have added the following sentence to the methods section: “*Because we are using log-log models: logYi = α + βlogXi + ε, we reported hazard ratios (HRs) with 95% confidence intervals (CIs) obtained by exponentiating Ln(2) times the estimated coefficients from the Cox model*.”

Regarding the linear regression analyses: Because we wanted to make the interpretation as easy as possible to describe in the results section, we chose to standardize the blood cell counts only to make results comparable and keep the rest of the variables untransformed. We have added this as a clarification to our methods section on page 14, lines 264-266: “To facilitate interpretation of these analyses, we chose to first standardize the blood cell counts only to make results comparable and keep the rest of the variables untransformed.”

13. *Paragraph 2, page 13- unclear if the results for blood cells and calcification volume are adjusted for anything.*

**Response:** We have now rewritten this section in order to avoid any confusion regarding the adjustments: “*Next, in order to explore possible mechanisms, we performed a cross-sectional analysis assessing the association of the granulocyte, platelet, and lymphocyte counts, and their derived ratios at baseline per standard deviation increase with calcification volume in each vessel bed (coronary arteries, aortic arch, extracranial carotid arteries, and intracranial carotid arteries) using linear regression models. To facilitate interpretation of these analyses, we chose to first standardize the blood cell counts only to make results comparable and keep the rest of the variables untransformed. We adjusted these analyses for age and sex (model I), and additionally for education, smoking status, BMI, diabetes mellitus, systolic blood pressure, diastolic blood pressure, blood pressure lowering medication, total cholesterol, HDL cholesterol and lipid lowering medication (model II).*” (page 14, lines 264-269).

14. *Table 1- include abbreviations for all variables (BMI, GLR, PLR, SII, etc.)*

**Response:** We have now included abbreviations for all variables in Table 1.

15. *Table 2- in caption it says “n= number of incident stroke events”, but 3 outcomes are presented above. What are the units for the HR? Is it per standard deviation, per doubling?*

**Response:** We thank the reviewer for this observation, and we have changed the definition of n to number of incident events instead of number of incident stroke events in table 2. Furthermore, we have included an interpretation for the HR in table 2 in the upper left corner: “*Per doubling of blood cell counts.*” (page 30).

**Reviewer #3**

*This is a nice paper on the links between the immune system and cardiovascular disease. The methods are pretty advanced, possibly more than absolutely necessary for valid estimates, and are carefully implemented. I do have a few concerns.*

1. *The biggest one is the mediation analysis. Mediation analysis has become popular but I think that it has some important limitations in this context. There is measurement error for both subclinical disease and immune cell subsets, both from the technique and the single time point (blood draws are done at a single time and some participants will have abnormal values at this time). Often this will weaken the associations between the three pieces of the mediation analysis (immune cells, subclinical disease and CVD outcomes). Since random error is put into the direct effect, it has a tendency to underestimate the mediation. Of course, measurement error is complicated, and it could also go in the opposite direction and inflate mediation estimates. At the least this needs to be discussed in the context of interpreting these results in the discussion.*

**Response:** We agree with the reviewer on the limitations of the mediation analysis (this point on the mediation analysis was also raised by reviewer #2, see our response on page: 05). We would like to emphasize that we have conducted this mediation analysis as a support and extension of our main analyses. Nevertheless, we have added the rightfully stated limitations to our discussion section: *“Finally, the measurement error for both subclinical disease and immune cell subsets limits the mediation analysis in its reliability. However, since random error is put into the direct effect, it has a tendency to underestimate the mediation. Of course, we cannot rule out that it could bias the results in the opposite direction and inflate mediation estimates..”* (page 22, lines 462-456).

2. *The use of joint models is nice to see. But I think that what the authors are doing is using a linear mixed model to estimate the change in immune cells over time, assuming a linear change over the time period (which seems to be 6 to 10 years given that visits are 3 to 5 years apart and some small number of participants have 3 blood draws). This isn’t a bad model and it will reduce measurement error quite a bit, but it still has some strong parametric assumptions that would make sense to explore. Can the authors provide any validation for the intuition of a linear model makes sense? What about using age as a proxy for time, and see if linear models work for the 3 to 5 year age bands? I know that with most participants having only two measurements options are limited, but when using such a cool and cutting edge technique it seems like a good plan to over-explain the assumptions, as readers will be less familiar with them.*

**Response:** We have added some explanation considering the assumptions of the joint models in response to reviewer #2. Considering the point of assuming linearity, we refer to our response to point 7 of reviewer #2. In brief: After careful examination of possible non-linearity, we found that log transforming our exposure variables provided the best solution to deal with non-linearity, due to the skewed nature of the exposure variables which untransformed could indeed cause problems by the extremes of the distribution. Further examination showed no highly non-linear longitudinal trajectories. Since we are adjusting for covariables and to enhance interpretability of our models, we continued with the log-transformed exposure variables, and keeping the models parsimonious without for example entering natural cubic splines. This reduces the risk of overfitting our models and convergence problems. We have added a phrase to the statistical analysis section to clarify this reasoning to the reader: “*Because the blood-cell counts and their derived ratios have a skewed distribution and to adhere to the linearity assumptions, the analysis was based on the natural logarithmic (Ln) transformation of their values*.” (page 12, lines 215-216). To further explain the adherence to the assumptions, we have also added to the methods section: “*We assessed the joint model assumptions by examining Cox-Snell residuals for the survival part and marginal residuals for the longitudinal part.”* (page 13, lines 241-242).

With respect to the strong parametric assumptions, we opted for a parametric but flexible specification of the baseline risk function, which we have now incorporated in the Methods section: “*For modelling survival data, we used a Cox regression model in which we include the true underlying profile of the blood cell counts as estimated from the longitudinal model. Here the baseline risk function is assumed piece-wise constant with six knots placed at equally spaced percentiles of the observed event times.*” (page 12, lines 223-225).

Regarding the comment about using age as a proxy for time; we reran the joint models using age as the timescale for the granulocytes and lymphocytes and risk of ASCVD, and found that the effects remained in the similar direction: HR 1.57, 95% 1.23-2.02 for granulocytes and HR 0.89, 0.73-1.07 for lymphocytes. We added these analyses briefly to our manuscript: “Lastly, we repeated analyses for the granulocytes and lymphocytes with risk of ASCVD using age as the timescale instead of follow-up time to account for potential residual confounding by age and to minimize potential effects of left truncation.” (page 13, lines 252-255) and in the results section: “Lastly, risk estimates were comparable when using the adjusted model and when using age as timescale instead of follow-up time.” (page 17, lines 323-234).

3. *The results themselves I have less to say about. Rotterdam is a famously high-quality cohort study with extremely well measured outcomes (and the definitions used were pretty typical). The measures of subclinical disease are well validated and high quality. The blood assays were done by a competent approach. I would have liked further subtyping of the immune measures, but the world is filled with questions where I wish there was more data. The effect estimates are compatible with existing literature and well reported. But high quality estimates like this are pretty novel. So I am generally pretty happy with the guts of the study.*

**Response:** We appreciate the reviewer’s points and further subtyping would definitely have been extremely interesting. Unfortunately we are limited by these crude measures of the immune system, as stated in our limitations section. We thank the reviewer for the positive and thoughtful feedback.

4. *Overall, I think a careful writing of the methods section to highlight the assumptions of the advanced approaches used would greatly enhance the interpretability of this manuscript for the target audience. If the authors are convinced that this would disturb the flow of the paper, at the very least a methods online supplement would be appropriate.*

**Response:** We thank the reviewer for these excellent suggestions and we have carefully added more explanation of the assumptions in the manuscript as a response to above points. We hope that this is satisfactory for the reviewer.
