# Supplementary material for: The association of innate and adaptive immunity, subclinical atherosclerosis, and cardiovascular disease in the Rotterdam Study: A prospective cohort study
Source: PLoS Med. 2020 May 7;17(5):e1003115. doi: 10.1371/journal.pmed.1003115 (PMC7205222; doi:10.1371/journal.pmed.1003115)
Supplement: Attachment — Submitted filename: Authors reply_20200410.docx [file pmed.1003115.s008.docx]

## Authors’ response to reviewers

**Manuscript title**: *Innate and adaptive immunity, subclinical atherosclerosis, and the risk of cardiovascular disease: a prospective cohort study*. Manuscript # PMEDICINE-D-19-03937R2

We have revised our manuscript according to the suggestions made by the editors and have addressed the final production issues. All revisions in the manuscript are indicated by their specific line numbers for your convenience (referring to the document with tracked changes).

**EDITORIAL:**

1. *Thank you for revising the title to add ‘Rotterdam’. Unfortunately, the study design has been lost and we need to add it back in. In addition, we have changed 'risk' to 'with association' as this is not a trial. As such please change your title to: Innate and adaptive immunity, subclinical atherosclerosis and association with cardiovascular disease in the Rotterdam study; a prospective cohort study*

**Response:** We have changed the title to the title as suggested by the editors (lines 1-2, page 1).

**FIGURES and GRAPHICS:**

1. *Please ensure that all main figures are referenced in the manuscript as Fig 1, Fig 2, etc. (including capitalization), rather than Figure 1, Figure 2, etc. Please note, however, that the file names themselves must not include the space, i.e. Fig1.tif, Fig2.tif, etc.*

**Response:** We have changed the reference to figure 1 on page 15 to *Fig 1* (line 286).

**ARTICLE FILE:**

1. *Please remove author degree titles from the byline of your article file.*

**Response:** We have removed ‘MD’ from line 5 page 1.

2. *The names for your co-authors Arfan Ikram and Kamran Ikram do not match across the article file and the submission form. Please ensure that all author names match verbatim, including the use of a middle name/initials and any special characters (accents, umlauts, etc.), as correct spelling and formatting of author names is vital for accurate indexing in PubMed.*

**Response:** Apologies, we have made sure that these names now match the article file and submission form.
